# Challenges and insights from implementing clinical decision support systems for radiologic imaging: experience from the MIDAS trial

**DOI:** 10.1186/s13244-025-02027-0

**Published:** 2025-07-05

**Authors:** Thomas Kroencke, Stijntje W. Dijk, Moritz C. Halfmann, Claudia Wollny, Joerg Barkhausen, Olav Janssen, Dimitris Rizopoulos, M. G. Myriam Hunink

**Affiliations:** 1 Department of Diagnostic and Interventional Radiology, University Hospital Augsburg, Augsburg, Germany; 2 Centre for Advanced Analytics and Predictive Sciences (CAAPS), University of Augsburg, Augsburg, Germany; 3 Department of Radiology and Nuclear Medicine, Erasmus MC University Medical Center, Rotterdam, The Netherlands; 4 Department of Epidemiology and Biostatistics, Erasmus MC University Medical Center, Rotterdam, The Netherlands; 5 Department of Radiology, University Medical Center Mainz, Mainz, Germany; 6 Department of Radiology and Nuclear Medicine, University Medical Center Schleswig-Holstein (UKSH), Lübeck, Germany; 7 Department of Radiology and Neuroradiology, University Medical Center Schleswig-Holstein (UKSH), Kiel, Germany; 8 Centre for Health Decision Science, Harvard T.H. Chan School of Public Health, Boston, MA United States of America

**Keywords:** Clinical decision support systems, Overdiagnosis, Referral, Healthcare costs, Radiology

## Abstract

**Abstract:**

Clinical decision support systems (CDSSs) have been developed to give guidance for referring physicians to make appropriate decisions at the point of care. The MIDAS study, a multicenter cluster randomized trial at four German university hospitals, was designed to evaluate the effectiveness of a CDSS for imaging referral (ESR iGuide) in routine clinical care. Based on our experience within the MIDAS study, we aim to describe the hurdles and difficulties, as well as the various insights gained, in the process of implementing a CDSS in a clinical and research setting. To successfully implement a CDSS for imaging requests, it is essential to monitor and address technical issues, adapt local workflows, define the scope and content, and prioritize user experience and acceptance.

**Critical relevance statement:**

By identifying and addressing the various technical, content-related, and workflow challenges, this article gives valuable insights to facilitate future implementations of the ESR iGuide and similar clinical decision support systems CDSSs for imaging orders.

**Trial registration number:**

Approval from the Medical Ethics Review Committee was obtained under protocol numbers 20-069 (Augsburg), B 238/21 (Kiel), 20-318 (Lübeck) and 2020-15125 (Mainz). The trial is registered in the ClinicalTrials.gov register under registration number NCT05490290.

**Key Points:**

- This manuscript reviews the challenges of implementing a clinical decision support system (CDSS) (ESR iGuide).
- Clinical implementation of a CDSS for imaging requests requires monitoring and adjustments in technical issues, local workflow, scope and content, and attention to user experience and acceptance.
- Our experience may equip stakeholders with the knowledge to proactively address these challenges.

**Graphical Abstract:**

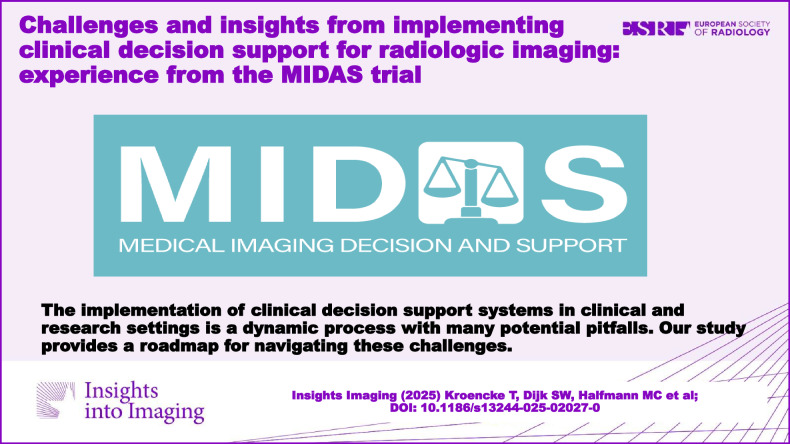

## Introduction

In 2014, the European Society of Radiology (ESR) conducted a survey on imaging referral guidelines and concluded that additional measures are needed to reinforce the use of such guidelines while at the same time strong support in the radiology community for the concept of integrating guidelines into clinical decision support systems (CDSSs) was noted [1]. Motivations to implement CDSSs range from the prospect of cost containment to the enhancement of the quality of care. Given that diagnostic imaging represents approximately 10% of total healthcare costs, and an estimated 20–50% of advanced imaging tests may not be of value to patients and may even confer a substantial risk due to ionizing radiation and contrast media administration. Therefore, strategies to optimize diagnostic test ordering have become a major area of interest [2–7].

The European Society of Radiology (ESR) iGuide is a CDSS originally based on the appropriateness criteria of the American College of Radiology (ACR), which was modified to account for the European context by including European guidelines. The ESR iGuide aims to cover 80% of requests in daily practice by reviewing clinical scenarios and indications, and it provides recommendations for appropriate imaging tests [8]. To enable point-of-care advice and feedback on ordering behavior, CDSSs are ideally deeply embedded in the computerized physician order entry (CPOE) environment of a hospital and seamlessly integrated into the workflow of ordering physicians.

We implemented the ESR iGuide in the context of the Medical Imaging Decision And Support (MIDAS) study, a multicenter cluster randomized trial at four German university hospitals designed to evaluate the effectiveness of a CDSS for imaging referral (ESR iGuide) in routine clinical care and gain insight into the tangible effects of CDSSs in a real-world setting [9]. The MIDAS study will broaden the evidence base for the use of clinical decision support systems for imaging referral and may serve as a reference point for the study of other CDSSs. This critical review aims to inform the reader about the challenges we faced on various levels during the implementation of the CDSS, give insight into common problems and solutions, and share our experience from conducting the MIDAS study. In doing so, we aim to equip policymakers, radiologists, and others considering CDSS implementation with the knowledge to proactively address these challenges and potentially enhance the usability of the iGuide.

## Materials and methods

The MIDAS study is a multicenter, cluster-randomized trial designed to investigate key outcomes associated with the use of a CDSS for image ordering. The main objectives are to determine key outcomes related to the appropriate use of diagnostic imaging tests and compare trends between departments randomized to the implementation of the active intervention (with CDSS) and the control condition (no CDSS). Details of the study protocol have been published elsewhere, and quantitative results of the trial are reported separately [9, 10]. Briefly, we recruited departments from four major German university hospitals for a multicenter cluster-randomized controlled trial with departments as clusters, combined with a before-after discontinued design. Departments (non-emergent, non-pediatric) expected to have a sufficient number of imaging requests for hospitalized patients were approached to participate.

In this report, we give an overview of the hurdles and challenges that we encountered in implementing the CDSS in clinical practice and in the MIDAS-specific research setting (Table 1). This study used the ESR iGuide to determine the appropriateness of imaging requests. The ESR iGuide is a CDSS for imaging referral guidelines developed by the European Society of Radiology (ESR) in cooperation with the American College of Radiology (ACR) and is based on published guidelines and the best available evidence for imaging. The methodology of the ESR iGuide development is described in detail in a paper by the ESR (2019) [11]. Based on ESR’s referral guidelines, appropriateness ratings are assigned to the clinical indications given and the examination ordered (Table 2). Physicians from participating departments were informed by local research teams about the purpose and design of the MIDAS study and the changes made to the CPOE system of the hospitals. Information sessions were held before data collection started and at the time of switching on decision support. In addition, users were given the possibility of providing electronic feedback about their experience with the system. During the conduct of the MIDAS study, the investigators from the participating centers held regular meetings to monitor the progress of the study and to keep each member of the consortium informed. Notes were taken and continually added to a living process document that captured the various challenges met by the study team and the decisions made to address these issues. Due to the different workflow architecture of the CPOE systems at participating centers, the study required the support of representatives of the vendors celsius37 and Dedalus as well as from the ESR iGuide support team and local system administrators. The study was further supported by a Data Safety Monitoring Board (DSMB) to whom study progress was reported. Finally, the heads from participating departments were involved in the study by providing consent for participation [12].

**Table 1:** Hurdles and challenges that we encountered during clinical implementation of the ESR iGuide and research-specific issues in the context of the MIDAS trial

Domain	Subtopic	Challenge	Solution
**Clinical implementation challenges**			
Workflow	CDSS-CPOE integration	Deep integration of the ESR iGuide into local CPOE required a procedure-driven workflow rather than an indication-driven workflow.	No alternatives were possible.
	CDSS	Optimal use of the system required structured order entry (rather than free text), which implies a rigid and predefined set of exams and indications/scenarios. Only a very low percentage of free-text imaging requests (5–10%) could be matched with iGuide recommendations.	Free text for indications was allowed in one hospital but discouraged (see item “lack of recommendation if free text is used”).
Content of the CDSS (ESR iGuide)	Protocolized setting	In highly protocolized settings, the iGuide was less helpful as the physician requesting imaging already knew what they wanted to request. For example, CT for pre-TAVR Workup.	Highly specialized examinations were not directed via the iGuide workflow.
	Re-naming indications	Translations from the original English were not always stringent and sometimes not directly linked to the terminology used in the German healthcare system.	The list of indications was extensively revised in terms of language.
	Missing indications	Several frequently used indications were not in the indication list provided by iGuide (e.g., post-operative follow-up). Physicians could therefore not always select the indication of choice.	Specific indications were added to the list.
	Lack of recommendations if free text is used	One hospital provided the option of entering indications as free text. Even when structured indications were available, the free text option was often used. This meant that the indication could not automatically be compared with the iGuide database, and therefore, no recommendation could be made.	We mapped additional terms as synonyms and motivated users through training to select an indication from the list that is nearest to what they intended to select. This did not lead to sufficient structured order entry: the integration of the CDSS was considered unsuccessful and this hospital was excluded from the trial.
	Scores for common oncological indications were missing	Not all combinations of indications and examinations lead to a recommendation; in particular, recommendations in the field of oncology were missing in the ESR iGuide.	A team of local experts revised the frequently selected oncological indications and provided appropriateness ratings. These new scores are available upon request.
	Local protocols and agreements	Some hospitals have local imaging algorithms that differ from those in the CDSS for specific indications.	We did not make local adaptations in order to keep study conditions the same
Training	User buy-in, acceptance and familiarity with the CDSS	Physicians were concerned about additional time needed to order imaging exams, limited choices and the possibility to track individual choices.	Concerns were addressed during pre-study training of participating physicians at the departmental level. A feedback option was installed in the user interface to allow instant feedback from ordering physicians.
	Staff fluctuation	High-volume ordering departments, such as the surgical emergency department, typically experience higher rates of staff turnover due to predetermined rotations of physicians	Trainings had to be offered repeatedly in order to reach all ordering physicians. Additionally, trainings were made available as on-demand resources via the respective intranet.
**Research-specific challenges**			
Eligibility	Inclusion and exclusion criteria	Departments needed to show that they could successfully integrate the ESR iGuide into their workflow prior to being considered eligible for randomization.	All departments from the hospital that were unsuccessful in integrating the ESR iGuide into the workflow were considered ineligible for participation in the trial.
Randomization	Matched departments	There was a discrepancy between which subspecialties fell under which department (e.g., internal medicine includes nephrology in one hospital but not the other), making a 1:1 matching of departments between hospitals impossible.	We randomized stratified for surgical vs. non-surgical specialty to preserve a balance between different specialty types.
	Burden without benefit	There was an additional burden associated with using the new system, while the control group did not receive benefits in the form of decision support.	We emphasized the importance and benefit of the trial, the future potential benefits, and the necessity of the control condition for a valid comparison to participating physicians.
Data collection	Inconsistencies in requests in the CPOE	Inconsistent request-indication sessions are unscored by the iGuide. For example, requesting an X-ray of the foot for the indication headache.	We explored the reasons for inconsistencies and resolved these where possible, and we educated users.
	Missing information in the CDSS	The ESR iGuide output does not automatically log information related to which department has sent the imaging request. While this is technically possible, data security regulations did not allow to transfer this information in order to keep information transfer to an external partner and the risk of a possible identification of patients at a minimum. Without this information, it would be impossible to analyze the data by department, which was our unit of randomization and observation. Individual departments could not receive feedback on the proportion of inappropriate imaging by their physicians from standard iGuide reports.	The missing department information could be retrieved from the Radiology Information System (RIS) data, partially for the initial months of the baseline data collection and fully during later months. The integration of the identifier caused the prolongation of the initial study phase.
	Incorrect department information in the RIS	The RIS report contains information about the department of the patients. The department information may be incorrect for some sessions, for example, when physicians order imaging while on consultation from a different department.	We accepted this as unavoidable and analyzed our results under the “intention-to-treat” analysis.
	Data protection and general data protection regulation	Concerns were raised by some of the sites about tracking the ordering behavior of individual physicians citing data protection laws.	Data protection is only an issue if physician-level or patient-level identifiable data is collected. The study protocol was specified to collect no information that could be traced back to specific individual patients or the requesting physician.
Merging datasets (CDSS-RIS integration issues)	Lack of a unique ID for each imaging request in the CDSS and RIS	We had difficulties merging the ESR iGuide data with RIS data due to a lack of a unique ID for each imaging request assigned to both datasets. Additionally, this was further complicated by sessions that had multiple indications for one imaging request or one indication for which multiple imaging requests were made.	The ID used by the iGuide was, after several months of efforts, also saved in the RIS data for each session. Prior to that, we were able to match most sessions in the two datasets based on the unique patient characteristics (age, sex), the indication and the modality requested.
	Timestamps in the CDSS and RIS	Matching missing session IDs using timestamps proved impossible because the two systems logged different times: ESR iGuide logs the time when the request is initiated to the iGuide system, and CPOE logs when the request is put through to the radiology department.	We did not use timestamps to merge the datasets. After negotiating the necessary updates by the vendor a unique session ID was added later in both datasets that could link the iGuide and RIS data.
	Missing sessions in the RIS	Some sessions were missing in the RIS dataset while registered in the ESR iGuide dataset. These were sessions closed in the CPOE system and therefore included in the RIS dataset of the next month.	Data were initially exported each month, and later for the entire study period, to avoid excluding otherwise valid sessions.
	Sessions with multiple exams in the RIS and CDSS	The unique Session ID was functional only for sessions with one exam.	A revision of the reporting system would have been necessary to obtain unique IDs for each exam, but due to the long processing time, and the potential resulting difference between baseline and implementation data, it was agreed to accept the loss of data where more than one exam was requested and leave these sessions as “invalid” as per the iGuide report.
	Multiple indications in the RIS and CDSS	For sessions with multiple indications, the iGuide internally selects one indication to best align with the clinical pathway. In the exported dataset, however, it was unclear which was chosen.	Sessions with multiple indications were excluded from the analysis. We accepted the small loss of data.
External factors	COVID-19 pandemic	The pandemic led to an increased workload and burden. Physicians were reluctant to make changes to their workflow in this period. IT departments were busy with other tasks.	Delayed implementation of the CDSS and start of the trial.
	Time to train physicians	Due to the time it takes to train departments and initiate the decision support, not all sites could start on the same date.	Analyzed data based on time since starting date, not the absolute date.

The columns “Domain” and “Subtopic” clarify whether the challenge is related to the CDSS itself, the CPOE, the RIS, the integration, or something else

Overview of the practical hurdles and challenges we encountered during the implementation of the ESR iGuide in routine clinical practice and the challenges specific to the research study setting. These challenges led to delays in the implementation and execution of the study, adjustments, newfound solutions, trade-offs, and compromises. In the text that follows the table, we highlight the most salient challenges and provide additional details

**Table 2:** Appropriateness categories and ratings in the ESR iGuide (cited from European Society of Radiology (ESR), Methodology for ESR iGuide content, ref. [8])

Appropriateness category name	Appropriateness rating	Appropriateness category definition
Usually appropriate (green)	7, 8, or 9	The imaging procedure or treatment is indicated in the specified clinical scenarios at a favorable risk-benefit ratio for patients.
May be appropriate (yellow)	4, 5, or 6	The imaging procedure or treatment may be indicated in the specified clinical scenarios as an alternative to imaging procedures or treatments with a more favorable risk-benefit ratio, or the risk-benefit ratio for patients is equivocal.
Usually not appropriate (red)	1, 2, or 3	The imaging procedure or treatment is unlikely to be indicated in the specified clinical scenarios, or the risk-benefit ratio for patients is likely to be unfavorable.

Summary of appropriateness ratings based on ESR’s referral guidelines. Appropriateness is rated ranging from 9 (highly recommended) to 1 (not recommended). A rating of 7–9 corresponds to “usually appropriate,” 4–6 is defined as “may or may not be appropriate,” and a rating of 1–3 is defined as “usually not appropriate.” The user is given the opportunity to adjust the requested imaging exam in response to the appropriateness rating and is provided with a list of exam types highlighting appropriate imaging exams for the indication entered in the system

## Results

**Fig. 1:**
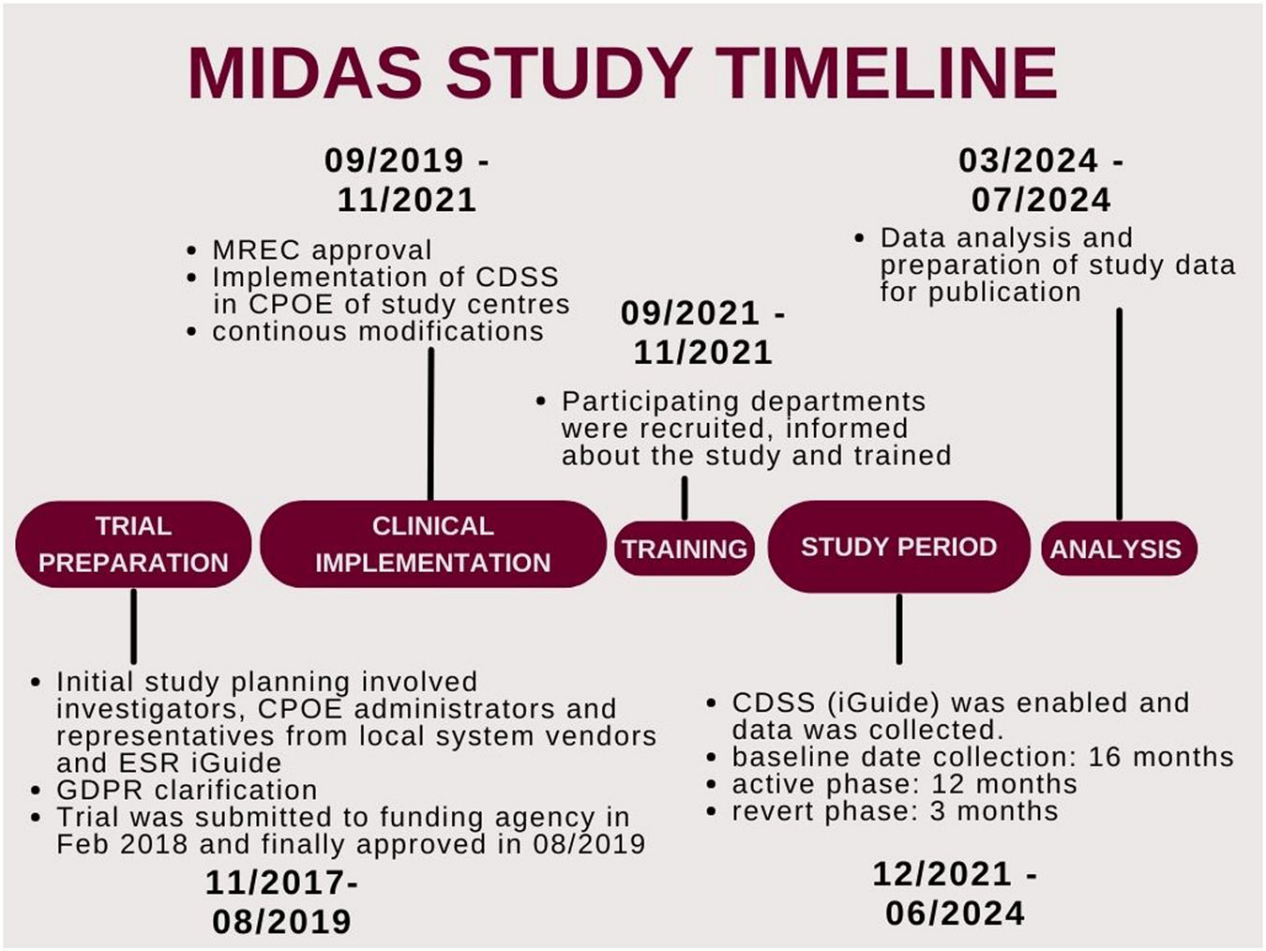
Timeline of the trial preparation, the clinical implementation of the ESR iGuide and the research study

Figure 1 illustrates the timeline of the trial preparation, the clinical implementation of the ESR iGuide and the research study. We had expected the clinical implementation to take 2 months, but in reality, it took 26 months. Additionally, the collection of the baseline data for the research study was extended and took a total of 16 months. After randomization and switching on decision support in departments assigned to the active intervention, data were collected for 12 months. In the revert phase, after removing decision support, data was collected for another 3 months.

## Challenges related to clinical implementation

### Workflow and CPOE

Computerized patient order entry (CPOE) can be defined as a stepwise process (workflow), which can be either procedure- or indication-driven. The procedure-driven workflow is initiated by the selection of an imaging exam as the first step from a predefined order list. Afterward, further specified information is given by selecting a clinical indication or scenario. The indication-driven workflow is initiated by selecting one clinical indication as a reason for requesting an imaging exam. Next, imaging exams can be selected by the ordering physician. Implementing ESR iGuide in the CPOE of all 4 participating hospitals required a procedure-driven workflow. Three hospitals required a structured order entry due to the requirements of the underlying CPOE system. This structured order entry consisted of a combination of a selected imaging exam, anatomic region, and indication using predefined catalogs out of which the ordering physician needed to choose. These drop-down lists in the structured order entry system were considered cumbersome to navigate.

One hospital had a preexisting CPOE system that allowed a free-text entry and enabled an elastic search for suitable indications. In this hospital, users performed an indication search before completing the free-text referral. The elastic search functionality was readily available and provided real-time suggestions from the iGuide catalog as the user entered their search terms. If none of the suggested indications were chosen by the user, they had the option to scroll to the bottom of the list and submit a free-text indication. Out of 42,855 sessions conducted between January 1st and July 16th, only 6767 (approximately 15.8%) were successfully scored, indicating significant limitations in the matching process. Additionally, the free-text input system, while flexible, posed challenges in terms of accurate and consistent matching with iGuide, particularly when clinical terminology varied widely or when insufficiently specific information was provided. Only a very low percentage of these free-text imaging requests (ranging from 5–43.8% between participating departments) could be matched with iGuide recommendations, leading to valid and scored sessions. Together with local representatives, the research team, vendors and the iGuide team, we attempted to increase the number of matched requests through training and instructing local physicians. Additionally, the most frequently entered free-text submissions within the system were rigorously checked for any errors leading to invalid and unscored sessions. Despite these efforts, the percentage of scored sessions among participating departments did not increase beyond 10%, on average; therefore, we decided to exclude the departments from this hospital and remove the CDSS.

The CDSS was integrated within the CPOE. While this was successful in general, limitations due to the vendor-specific requirements needed to be addressed. Although we intended to have a user-friendly order entry, the requirement to use predefined lists and the limited space to display given selections was a major issue. In addition, any changes needed to be negotiated with the vendors and were met with significant delays due to the vendors’ timelines of remote fixes and system updates. Furthermore, the vendor-specific CPOE did not allow modification of the process by, for example, providing a list of very frequently selected indications in the form of a Top 10 list at the department level, which could have improved user-friendliness.

### Content

While the catalog of imaging exams at each participating hospital remained unchanged during the time of the study, the selection of a predefined indication was a new element in the ordering workflow. Several issues were brought forward by physicians, mainly related to the content and display of the list of indications and clinical scenarios:

- Frequently ordered imaging requests for either planning of a treatment (e.g., CT angiography for transcatheter aortic valve replacement (TAVR) planning) or imaging confirmation of a treatment (e.g., imaging after fixation of a fracture) were considered highly protocolized scenarios that did not require decision support.
- While the iGuide’s list of selectable indications/clinical scenarios is extensive, it is still incomplete, and some indications did not match clinical routine. Missing indications led to a lack of a recommendation from the CDSS. As an indication of the size of this problem, of the nearly 129,000 valid requests captured by the iGuide from the three hospitals included in the final analysis of the MIDAS trial, 48% did not receive an appropriateness score.
- In particular, recommendations and appropriateness scores for oncological indications related to staging, monitoring and follow-up were initially missing from the ESR iGuide (whereas diagnostic indications were included). A small working group was urgently formed, which reviewed German national and international guidelines (European Society for Medical Oncology and National Comprehensive Cancer Network guidelines) and defined missing appropriateness scores based on this additional evidence.
- The translations of the available indications had to be extensively revised, as the choice of words was not always stringent, and the terminology of the German healthcare system was not always used.

### Training of participating departments and physicians

Physicians of participating departments underwent a training session, which included a presentation of the study goals, the study-related changes in the workflow in imaging ordering and a questions-and-answers session to address concerns and enhance buy-in from users. A short video clip illustrating the workflow was made available on the local electronic information board of each hospital. In addition, an option to give feedback directly at the time of using the CDSS was installed, and this information was continuously collected, analyzed and, where appropriate, changes were made if possible.

## Challenges specific to the research setting

### Randomization by department

The MIDAS study was designed as a multicenter study involving clinical departments from the university hospitals Augsburg, Kiel, Lübeck and Mainz. We assumed that these hospitals have similar departments and services. Our initial plan was to match specific departments, e.g., Internal Medicine from one hospital with another hospital. Two challenges arose: (1) Not all departments from all hospitals signed up to the study, and (2) there was a discrepancy between which subspecialties fell under which department (e.g., Rheumatology and Nephrology were part of one Internal Medicine department in one hospital and were assigned to separate departments in another hospital). Therefore, we decided to randomize stratified for surgical vs. non-surgical specialty instead, to still preserve a balance between different specialty types.

### Burden without benefit for the control group

Participating departments benefited only in part from decision support throughout the study, since in the control group, it was mandatory to use structured order entry for a designated time while the CDSS was turned off. In a highly demanding work environment, every additional step in the ordering process is perceived as time-consuming and non-beneficial from the perspective of the ordering physician. To ensure adherence to the study workflow, we made efforts to inform all participating departments about the requirement of a control group within the study and highlighted the benefits that may result in general from such a study.

### Inconsistencies, missing and incorrect information

In most requests that are processed via the CDSS, a meaningful combination of an ordered imaging examination and indication is entered. Nevertheless, some combinations may be unforeseen or illogical and therefore they are not matched by the CDSS, resulting in non-scored imaging requests. We tried to identify these combinations, solved them on a case-by-case level and educated users to avoid non-scored requests. As the study relied heavily on the correct identification of ordering at the departmental level, it was of utmost importance that the individual imaging order was placed strictly through access of the CPOE at the departmental level. However, order entries may be pre-filled after consultation from another department. This may have resulted in the assignment of an order to an incorrect department. We accepted this as unavoidable and analyzed our results under the “intention-to-treat” analysis. A department was considered as an intervention or control cluster based on the assigned department name. This did result in some sessions in the control condition having the request changed after CDSS feedback. It should be emphasized that most of these problems arose as part of the user interaction, workflow and CPOE integration (front-end) since the CDSS (backend) is dependent on the information transferred.

### Data protection and general data protection regulation

During information and training events, the question came up whether the ordering behavior of individual physicians would be trackable through the system, leading to monitoring of individual decisions with possible dire consequences. The study protocol was revised to specify that only anonymized data is collected at the session level. All collected data could be traced to specific departments, but not to specific patients or physicians. This, however, also meant that sessions were assessed as standalone sessions, no conclusions could be drawn for specific patient pathways (e.g., whether for one patient two CTs in 1 week are appropriate) or from individual physicians (e.g., are junior staff more likely to request inappropriate exams?).

### Merging datasets

Several issues arose regarding the matching of data entered in the CPOE to data entered in the ESR iGuide. The transfer of data between the CPOE and the CDSS, which is hosted on an external web server, was kept to a minimum due to safety data regulations. Moreover, the particular datasets in the hospital CPOE contained information that was needed for the trial but were not available in ESR iGuide. This made it necessary to match the information from the two systems in order to obtain all the necessary data for the analysis. Simply, matching requires a biunique identification of data entry and corresponding assignment of an appropriateness score. A unique ID connecting these two data sources was not available at the beginning of the study period. This delay, however, resulted in a loss of approximately 30% of the baseline data when the originating department could not be inferred from RIS sessions with the same unique combination of age, sex, hospital, indication, and exam. The time-stamp could not be used to link unique sessions, as the time-stamp in the RIS data was based on session initiation, and the ESR iGuide data the time of completion. Also, the CPOE allowed orders that included multiple imaging requests under the same order number. In an early phase with ID, this was unfortunately used for all requests for an order. This was later corrected. A pilot study could have helped to identify this before the actual data collection had commenced.

### External factors

The COVID pandemic delayed the implementation and the trial. During this time, which was while we were initiating our study, physicians were burdened and overwhelmed with taking care of COVID-19 patients. A change in workflow and training physicians to adhere to the structured order entry of the CPOE and work with the CDSS was simply not acceptable. Within the context of pandemic preparedness and to address similar challenges in future implementations, it might be reasonable to prepare a pilot study for training and analysis of common and system-relevant challenges. The planning process, the implementation of the plan, the testing of the plan, and the revision of the plan all serve an important purpose in allowing key stakeholders to become familiar with the issues at hand.

## Discussion

### Summary of findings

Our study meticulously documented the implementation process of the ESR iGuide CDSS within a real-world, multicenter clinical and research setting, the MIDAS trial. The process was longer and more complex than anticipated, highlighting the numerous challenges inherent in integrating new tools into established clinical workflows. While the ESR iGuide offers comprehensive decision support for a wide array of imaging requests, we encountered hurdles related to workflow adaptation, content gaps (particularly in oncology), and local order entry system variations. In the clinical setting, these challenges impacted our timeline, user experience, and percentage of sessions that were scored and valid. The research setting presented unique challenges related to randomization, data collection, and ethical considerations. These findings underscore the importance of thorough planning and adaptability when implementing CDSSs in both clinical and research contexts.

### Comparison to existing literature

Our findings resonate with previous studies evaluating the effectiveness of such systems, particularly regarding user experience and implementation of a CDSS for imaging requests in clinical practice. Similarities can be observed between the challenges we faced with the implementation of the CDSS into local CPOE systems and those in the Medicare Imaging Demonstration (MID) project. One prominent finding highlighted in the Report on Pre-Implementation and Implementation Experience of MID was that “the integration / interoperability of DSS with EMRs (Electronical Medical Records) was more challenging than expected and resulted in delays in launching the demonstration” [13, 14]. The MID conveners estimated that the time needed to implement a nationwide DSS could range from 6 months to 4 years. However, they generally considered that 1–2 years would provide sufficient time to implement the DSS. This time frame matches our experience and highlights the complexity of integration. Interestingly, some specific problems came to light in the MID as well as in our study: a large percentage of imaging orders could not be analyzed because they were not covered by guidelines, the common use of order sets (e.g., combinations of imaging exams) resulted in duplicates, imaging orders were placed by proxies rather than physicians, and users defaulted to an easy text entry rather than picking from the limited list of indications/scenarios which resulted in unscored orders [13, 14]. With respect to our study, of the nearly 129,000 valid requests captured by the iGuide from the three hospitals included in the final analysis of the MIDAS trial, 48% did not receive an appropriateness score [10]. This may have among others these following particular reasons: none one of the > 15,000 appropriate use criteria selection options that are part of the ESR iGuide catalog fit to the request, a high percentage of unscored indications corresponding to standardized pre- or post-operative follow-up imaging that were added to a list of selectable indications outside of the ESR iGuide, errors that have been made in the order entry.

A limited number of trials employed the ESR iGuide in comparable settings but did not specifically report their experience implementing the CDSS or restricted the application of ESR iGuide to certain imaging modalities or indications. In a retrospective study, Rosen et al examined the appropriateness of CT examinations employing the ESR iGuide for clinical decision support. A substantial rate of CT examinations ordered was classified as inappropriate for a given indication, and it was found that inappropriate exams are related to physicians’ specialty and seniority [15]. They found that exams ordered by surgeons were considered appropriate to a higher rate than those ordered by non-surgical disciplines and attributed this to rather defined clinical questions faced by surgeons as opposed to diagnostic challenges common in internal medicine. To some extent this is in line with our experience in the MIDAS study: especially those disciplines with a predefined workflow that implied specific imaging tests (e.g., CT imaging prior TAVR, post-operative x-ray) requested adjustments to streamline recurring clinical scenarios. This observation is further supported by published experience from the MID, where conveners noted that lessons learned from the MID was the design of a CDSS may need to differ between generalists and specialists, arguing “that generalists may prefer to begin the ordering process from the point of patient symptoms, whereas specialists prefer to start with specification of the imaging procedure” [13].

User acceptance is critical for the acceptance of a CDSS for imaging referral, and we tried to address concerns regarding additional time needed for navigating the CDSS, disrupted workflows, limited choices and data protection by offering training and feedback. Singer et al assessed the use of the ESR iGuide as a web-based reference tool with regard to acceptance by physicians using a voluntarily submitted post-intervention questionnaire [16]. Although not directly comparable to a CPOE integrated use of iGuide and based on a very limited number of responses, they identified dissatisfaction of users with key areas of the CDSS, such as meaningful decision support, the evidence basis of recommendations, and overall confidence in imaging decision support. While a majority of physicians found the system user-friendly, the various functions were considered not well integrated. Overall, the majority of physicians stated that they do not want to use the CDSS more frequently or recommend it to colleagues, highlighting that despite benefits, several important aspects influence user acceptance.

### Strengths

The strengths of our study lie in its comprehensive documentation of the ESR iGuide CDSS implementation process within a real-world, multicenter research setting. This approach provides valuable, practical insights that can inform and potentially streamline future implementation endeavors. Notably, the materials developed during this study, such as the supplementary recommendations for oncological indications providing scores for previously unincluded indications, offer a tangible resource to enhance the existing iGuide. By embedding our study within the actual CPOE systems of participating hospitals, we gained a nuanced understanding of the real-world challenges and complexities of CDSS implementation, surpassing the limitations of isolated, controlled settings. This real-world perspective could be valuable for healthcare institutions and researchers seeking to implement and evaluate CDSSs in clinical practice. Furthermore, our multicenter design, encompassing diverse hospital environments, enhances the generalizability of our findings, offering a broader understanding of the challenges and solutions applicable to various contexts.

### Limitations

However, our study is not without limitations. We encountered significant challenges in integrating the ESR iGuide into one of the four intended sites, underscoring the difficulties in achieving universal compatibility with all CPOE systems. Of note, ESR iGuide works mainly as a backend service when integrated and relies on the granularity of data provided by the respective CPOE. This led to problems in the research context of the MIDAS study. These limitations highlight the need for continued efforts to improve CDSS adaptability and integration across diverse healthcare infrastructures. Additionally, our study’s focus on larger departments may have resulted in an oversight of unique challenges faced by smaller units. Moreover, the context-specific nature of our study, including challenges related to the COVID-19 pandemic and data collection delays, means that our timeline should not be considered a blueprint for future projects. Rather, it serves as a transparent account of the hurdles we encountered, potentially aiding future endeavors in anticipating and mitigating similar obstacles. The MIDAS trial was set in academic hospitals with specialized departments and tailored referral patterns. The implementation of a CDSS may pose different challenges in primary care settings, non-academic hospitals, or hospitals with less specialized departments. Furthermore, the implementation of the iGuide can be done via a web-based client, which would allow a more flexible approach than an implementation in a rather static CPOE system like the one present at the four academic hospitals of the MIDAS study. Consequently, other settings may face other challenges and demonstrate different results.

Despite these limitations, our study offers valuable contributions to the field. The identification of preventable challenges, such as the missing integration of a session ID for department-level analysis, can inform future research and clinical implementations, potentially saving time and resources. Furthermore, our need for department-level appropriateness data could inspire the incorporation of such features into standard iGuide reports, enhancing their utility for targeted department-level training and research initiatives.

### Future directions and recommendations

The implementation of CDSSs in clinical and research settings is a dynamic process with many potential pitfalls. Our study provides a roadmap for navigating these challenges, offering recommendations to improve the implementation process:

- **Tailored implementation:** Future implementations should prioritize a thorough assessment of existing workflows and tailor the CDSS integration to minimize disruptions and maximize user acceptance. Important steps in doing so include reducing the number of unscored sessions and ensuring the process of selection of indications and exams is intuitive and fast. Future research could include further qualitative reflections on barriers faced by users to further develop the tool.
- **Comprehensive training and support:** Comprehensive training programs and ongoing support are essential to ensure that users understand the CDSS’s functionality and can effectively incorporate it into their practice.
- **Continuous refinement:** CDSS implementation should be viewed as an ongoing process, with continuous evaluation and refinement based on user feedback and evolving clinical needs.
- **Research-specific considerations:** Studies involving CDSSs should consider running smaller pilot programs to ensure that all required data is collected and accurately exported from the CDSS system.

By addressing these challenges and adhering to these recommendations, future implementations of CDSSs like the ESR iGuide can be optimized to enhance their potential for improving clinical decision-making, research and patient care.

## Conclusion

In conclusion, our study provides a comprehensive and transparent account of the real-world challenges and opportunities encountered during the implementation of the ESR iGuide CDSS. By identifying and addressing the various technical, content-related, and workflow challenges, we offer valuable insights to facilitate future implementations of this and similar CDSSs in diverse healthcare settings. While CDSS technology holds promise in aiding physicians with imaging decisions, our findings underscore the need to acknowledge and proactively address the complexities of implementation and the potential burdens on physicians. This review highlights the necessity for additional research to investigate the potential of CPOE with CDSS in different settings, encompassing process and patient outcomes, and to ascertain optimal practices to overcome the obstacles to its wider implementation. Ultimately, our goal is to contribute to the ongoing efforts to harness the potential of CDSSs in improving clinical decision-making, optimizing imaging appropriateness, with the objective of improving patient care.

## Data Availability

Quantitative results of the RCT and additional data have been reported (Dijk et al [10]).

## Abbreviations

ACR: American College of Radiology
CDSS: Clinical decision support system
CPOE: Computerized physician order entry
CT: Computed tomography
ESR: European Society of Radiology
ESR iGuide: ESR imaging referral guidelines
MID: Medicare imaging demonstration
MIDAS: Medical imaging decision and support
RIS: Radiology Information System
TAVR: Transcatheter aortic valve replacement
